# Examination of Solvent Interactions with Trp-Cage in 1,1,1,3,3,3-Hexafluoro-2-propanol-water at 298 K through MD Simulations and Intermolecular Nuclear Overhauser Effects

**DOI:** 10.1021/acs.jpcb.3c01029

**Published:** 2023-05-30

**Authors:** J. T. Gerig

**Affiliations:** Department of Chemistry & Biochemistry, University of California, Santa Barbara, Santa Barbara, California 93106, United States

## Abstract

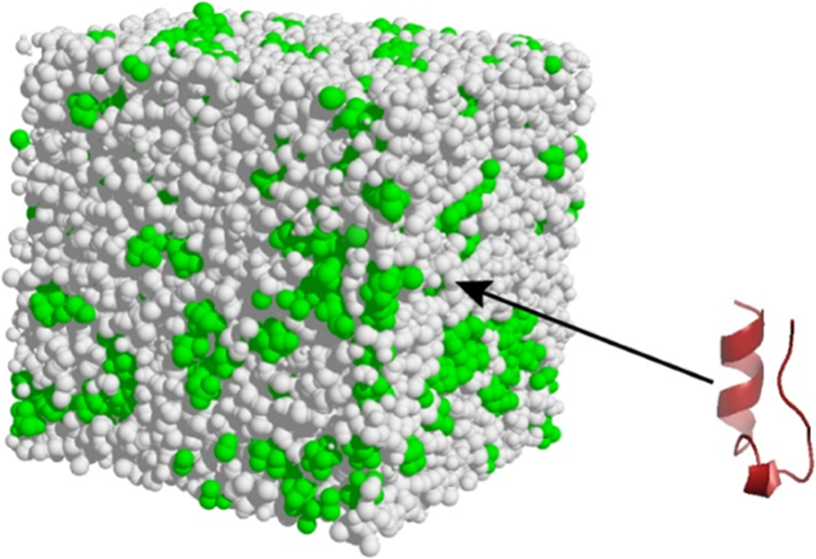

MD simulations of the peptide Trp-cage dissolved in 28% hexafluoroisopropanol (HFIP)-water have been carried out at 298 K with the goal of exploring peptide hydrogen–solvent fluorine nuclear spin cross-relaxation. The work was motivated by the observation that most experimental fluoroalcohol-peptide cross-relaxation terms at 298 K are small, both positive and negative, and not always well predicted from simulations. The cross-relaxation terms for hydrogens of the caged tryptophan residue of Trp-cage are substantially negative, a result consistent with simulations. It was concluded that hexafluoroisopropanol interactions near this part of the peptide are particularly long-lived. While both HFIP and water are present in all regions of the simulation box, the composition of the solvent mixture is not homogeneous throughout the system. HFIP generally accumulates near the peptide surface, while water molecules are preferentially found in regions that are more than 1.5 nm from the surface of the peptide. However, some water remains in higher-than-expected amounts in the solvent layer surrounding 6Trp, 9Asp, Ser13, and Ser14 residues in the helical region of Trp-cage. As observed in simulations of this system at 278 K, HFIP molecules aggregate into clusters that continually form and re-form. Translational diffusion of both HFIP and water appears to be slowed near the surface of the peptide with reduction in diffusion near the 6Trp residue 2- to 3-fold larger than calculated for solvent interactions with other regions of Trp-cage.

## Introduction

Small, water-soluble, fluorinated alcohols such as trifluoroethanol (TFE) and hexafluoroisopropanol (HFIP) have long been known to have significant influences on the conformations of peptides and proteins in aqueous solutions.^1,2^ Typically, the presence of these materials favors the formation of helical structures of peptides and the modification of secondary and tertiary structures in proteins. The effects observed in a particular system may derive from the enhanced acidity of the fluoroalcohol, influence of the fluoroalcohol on hydrogen-bonding or hydrophobic interactions, aggregation of the alcohol, and preferential interactions with the polypeptide. The aggregation behavior of a protein or peptide may be altered as a result of the influences of a fluoroalcohol. There is evidence that TFE, and presumably other fluoroalcohols, can be peptide structure-enhancing through the self-aggregation of the alcohol and preferential interactions with peptide structures.^3^ Peptides dissolved in solvents containing trifluoroethanol in water have been widely studied and the mechanisms that have been proposed to explain how the presence of TFE alters peptide conformation explored by experiment and molecular dynamics simulations.^1,3−10^

Protein synthesis, modification, and folding are highly regulated in cells.^11^ Understanding the processes by which a peptide or protein folds to a biochemically relevant structure in a specific context remains a vigorous area of research despite the availability of computer programs that can reliably predict the three-dimensional structures of these molecules.^12^ Small peptides are essential to such efforts in that, while interesting in their own right, they provide model systems that can be investigated in detail by a wide variety of spectroscopies.

Perhaps the best-known peptide or “miniprotein” in this context is Trp-cage (NLYIQ QLKDG GPSSG RPPPS), a 20-residue peptide that takes up a compact structure in water that features a hydrophobic core, a short α-helical region, and a polyproline II region.^13^ In water, the peptide folds to this structure about 4 μs at 296 K.^14^ Despite many efforts, the process(es) producing folding of Trp-cage remains controversial, with some experimental approaches indicating a simple equilibrium between folded and unfolded forms of the peptide is present while other methods suggest the presence of intermediates.^15,16^

Interactions with solvent molecules are critical to defining the three-dimensional structures and structural dynamics of a protein. Using a mixture of water and a small, water-soluble organic molecule as a solvent for a protein system provides an avenue for altering the structure of the protein and dynamics associated with it. Thus, phenomena such as protein folding or enzyme activity can be altered by the nature and amount of organic co-solvent present. There is much evidence that organic co-solvents can interact preferentially with proteins in a site-specific manner;^17,18^ identification of the sites of interaction can provide leads to interaction sites for drugs.^19^

This laboratory has been interested in examining the conformational effects of fluorinated alcohols on peptides and proteins by exploiting the advantages of fluorine NMR spectroscopy.^20−22^ The primary tool has been the study of intermolecular nuclear Overhauser effects (NOEs) arising from interactions of fluoroalcohols with peptides.^23−26^ An intermolecular NOE is characterized by a cross-relaxation parameter ∑_XY_ which arises from magnetic interactions between spin X of the solute and spin Y of the solvent. Theory such as that due to Ayant et al.^27^ shows that ∑_XY_ depends on the gyromagnetic ratios of spins X and Y, the concentration of solvent spins, the distance of closest approach of the molecular entities holding spins X and Y, and the mutual translational diffusion coefficient of solvent and solute.^28^ Exceptions to the predictions of theory may signal chemical interactions of solvent with the peptide that are more complex than those envisioned by the theory, including the presence of interactions that are longer-lasting than simple collisions.

**Figure 1:**
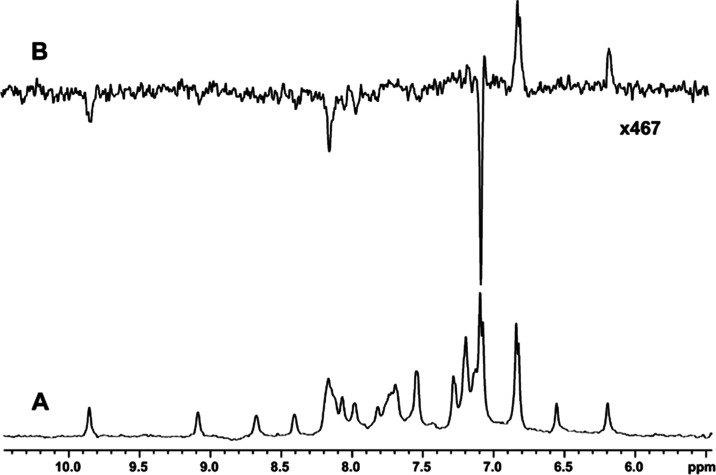
Peptide proton-solvent fluorine intermolecular NOEs for Trp-cage in 30% HFIP-water at 298 K and pH 7. Spectrum A is a control spectrum, while spectrum B shows the NOEs detected in the low-field portion of the spectrum at a mixing time of 400 ms. NOEs for the upfield region of the spectrum, shown in the Supporting Information of ref (26), are a mixture of positive and negative effects. Reproduced from Ref (26). Copyright [2006, American Chemical Society].

The conformation of Trp-cage dissolved in 30% HFIP-water at 278, 298, and 318 K is virtually the same as the structure found in water.^26^ A study of fluoroalcohol-peptide NOEs that arise between Trp-cage hydrogens and the fluorines of HFIP in 30% HFIP-water at 278 K showed that MD simulations of the system reliably predict many peptide hydrogen–solvent fluorine cross-relaxation terms to within the estimated experimental uncertainty.^22^Figure 1 shows experimental HFIP fluorine-Trp-cage hydrogen NOEs observed at 298 K. In contrast to what was generally observed at the lower or higher temperatures, at 298 K many of the HFIP-peptide hydrogen intermolecular NOEs are not detectable above the noise level of the experiment. At 298 K, observable NOEs were often relatively small in magnitude and of either algebraic sign. In contrast, strongly negative peptide–solvent NOEs were observed for some protons of the 6Trp residue (Figure 1). Preliminary investigation showed that some observed NOEs were not in agreement with predictions from MD simulations of this system. It was hoped that further work would indicate the origins of disagreements between experiment and the calculations.

In what follows, we have used the symbol ∑_HF_ rather than the conventional symbol (σ_HF_) to represent the cross-relaxation rate produced by the interaction of solvent fluorine spins with peptide hydrogens. The symbol σ_HF_ will be reserved for the collision diameter parameter used in the Lennard-Jones 12-6 function representing the nonbonded interaction of a solvent fluorine with peptide hydrogen.

## Experimental Section

### Experimental Cross-Relaxation Parameters (∑_HF_)

Studies of cross-relaxation between HFIP fluorines and hydrogens of Trp-cage dissolved in 30% HFIP-*d*_2_-water (v/v) at 298 K have been previously reported.^26^ Many experimental peptide–solvent CF_3_ cross-relaxation parameters are small and could not be estimated with high accuracy due to spectral noise; practical considerations such as available instrument time and unavoidable instrumental drifts limited the S/N improvements that could be achieved by signal averaging. All of the previously reported experimental results were reconsidered for the present work, and a few ∑_HF_ parameters have been updated. It is estimated that the uncertainties of ∑_HF_ parameters reported range from 10 to 30% depending on the S/N ratio.

### Starting Structures for Simulations

Starting coordinates for Trp-cage were those of the first model given for PDB structure 1L2Y (www.rcsb.org/pdb/explore/explore.do?structureId=1l2y).^13^

### Molecular Dynamics (MD) Simulations

All simulations were done with GROMACS (Versions 5.1-2 and 2016.4)^29^ running locally on a CurrentBuild Computer WS2 workstation or on the COMET system at UC San Diego, then part of the NSF-sponsored XSEDE consortium. For most work reported here, a cubic simulation cell 6.7 nm on a side containing 1 Trp-cage, 482 HFIP-*d*_2_, 7434 water molecules, and a chloride ion was used. It should be noted that the mole fraction of HFIP in this system was 0.061 while the mole fraction of HFIP in the comparison experimental system was 0.068. It is believed that this difference does not affect the conclusions reached. A few simulations were run with simulation boxes 8.35 and 5.25 nm on a side. Results from these are given in the Supporting Information.

Force field parameters used were the same as those described previously for the study at 278 K.^22^ Additional details regarding the force field used are provided in the Supporting Information. The tertiary and hydroxyl hydrogens of HFIP were present as deuterium. The TIP5P-Ew water model was used.^30^ The combination rule for σ nonbonded parameters was the arithmetic mean () as used in the AMBER force fields while the combination rule for ε_*ij*_ nonbonded parameters was the geometric mean (ε_*ij*_ = (ε_*ii*_ε_*jj*_)^1/2^).

Periodic boundary conditions were applied and motion of the model center of mass was corrected at each step during the calculations. It was assumed that artifacts arising from the periodic boundary conditions and the finite size of the simulation cell were negligible.^31^ Covalent bonds of the peptide and HFIP were constrained to constant length by the LINCS procedure^32^ while the SETTLE algorithm was used to constrain bond lengths of water.^33^ Sample temperatures were regulated by velocity rescaling with a coupling constant of 0.1 ps.^34^ Pressure was maintained at 1 atm by use of the Berendsen pressure coupling method^35^ with a coupling constant of 1 ps. The particle mesh Ewald (PME) method for long-range electrostatics was used.^36^ Cut-offs for electrostatic and van der Waals terms were 1.4 nm. The integration time step was 0.002 ps. Snapshots of the system coordinates for analysis of solvent interactions were usually taken at 10 ps intervals. Random initial atom velocities were assigned using a different seed for each simulation; systems were equilibrated for times ≥2 ns before production runs were initiated. Data reported in this paper are the average of results from 5 to 10 independent trajectories.

### Analyses of Molecular Dynamics Trajectories

Programs contained within the GROMACS package were used to compute the system density and self-diffusion coefficients of solvent components *via* the Einstein relationship.^29^ The peptide diffusion coefficient was estimated from the observed displacement of the peptide over the course of the 1000 10 ps intervals. Locally developed programs were used to obtain internuclear distances as a function of time, estimate durations of solvent interactions, count local populations of solvent molecules, and estimate local diffusion coefficients of solvent molecules as a function of distance from a specific peptide hydrogen. Computation of the cross-relaxation parameter ∑_HF_ that describes dipole–dipole relaxation of a peptide hydrogen by a group of identical solvent *F* spins followed the procedure described previously.^22,37^ In the present work the correlation function, defined in previous papers, was typically represented by 1000 points obtained in calculations that averaged 19 000–59 000 evaluations of each time point. Correlation functions were fit to a sum of exponential functions using a local version of Provencher’s program DISCRETE.^38^ (see http://s-provencher.com/index.shtml.) The optimum fit typically used four or five exponential terms. For the present systems at 298 K, the expression for ∑_HF_ often led to a relatively small value for ∑_HF_ as a difference between two larger quantities.^39^

MD simulations must reproduce several chemical phenomena in this system if they are to lead to calculated intermolecular NOEs in agreement with experimental results. These include the conformational dynamics of Trp-cage and the dynamics of interaction of each solvent component with the peptide and with each other. The time scales for these may range from seconds to picoseconds. Braun and Steinhauser have pointed out that relatively long simulations might be required to produce reliable estimates of intermolecular cross-relaxation terms.^40^ In an attempt to obtain ergodic results in a practical amount of time,^41^ we averaged the results of up to 10 independent simulations of 0.1–0.6 μs duration each in the hope that the considerations indicated will be sufficiently accounted for.

## Results

### Computed System Densities and Translational Diffusion Coefficients

The intermolecular NOE depends on the diffusion of the peptide and the solvent components. Table 1 compares experimental density and translational diffusion data for the present system calculated from simulations as described earlier. System density was reproduced well by the simulations while peptide and fluoroalcohol diffusion coefficients calculated from the MD trajectories were within 5% of the experimental values for this system. As is usually observed in similar fluoroalcohol–water systems, the diffusion coefficient for water is less well predicted by the simulations, being overestimated by 42% in this case.

**Table 1:** Properties of Trp-Cage-HFIP-Water System at 298 K

solute	calculated	exp.
cell edge, nm	6.695	
number of HFIP	482	
number of water	7434	
mole fraction HFIP	0.061	
density, kg m^–3^	1207	1215^a^
*D*_Trp-cage_, m^2^ s^–1^ × 10^10^	0.84 ± 0.05	0.82^b^
D_HFIP_, m^2^ s^–1^ × 10^10^	4.8 ± 0.2	4.5^b^
*D*_H_2_O_, m^2^ s^–1^ × 10^10^	17. ± 0.1	12^b^

a Estimated by extrapolation of data for the undeuterated system provided by Yoshida et al.^42^

b Experimental data are from Chatterjee and Gerig.^26^

### Aggregation of HFIP in Water

**Figure 2:**
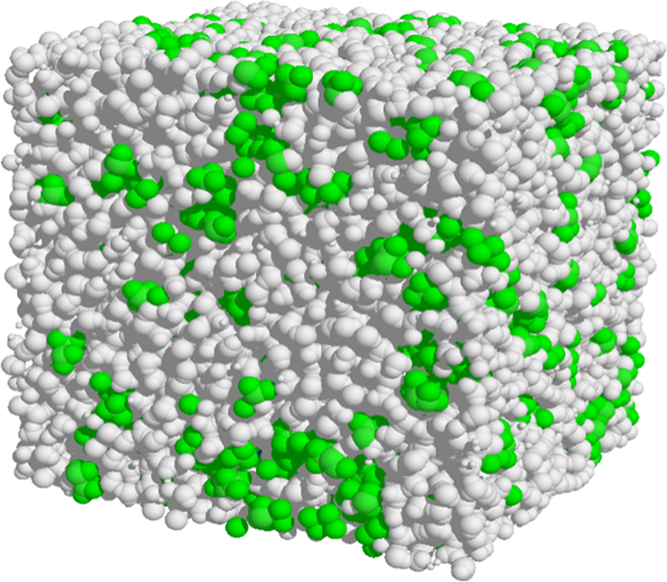
View of simulation box used in the present work. The cell is 6.69 nm on an edge and contains 482 molecules of HFIP (green), 7434 water molecules (gray), a molecule of Trp-cage, and a chloride ion. The latter two are near the center of the box and not visible in this view.

HFIP is significantly aggregated in aqueous solutions, with maximum aggregation taking place near 30% HFIP in water.^24,43−45^Figure 2 shows a view of the simulation box used in the present work. It confirms that the force field used predicts aggregation of HFIP molecules in 28% HFIP-water at 298 K. Several simulations of 0.2 μs duration indicated that, on average, about 62% of HFIP molecules are present in aggregates of up to 16 molecules. More fluoroalcohol aggregation is expected at lower temperatures; the same analysis of simulations at 278 K indicated that 89% of HFIP is present in aggregates.^22^

### Conformational Variations of Trp-Cage

**Figure 3:**
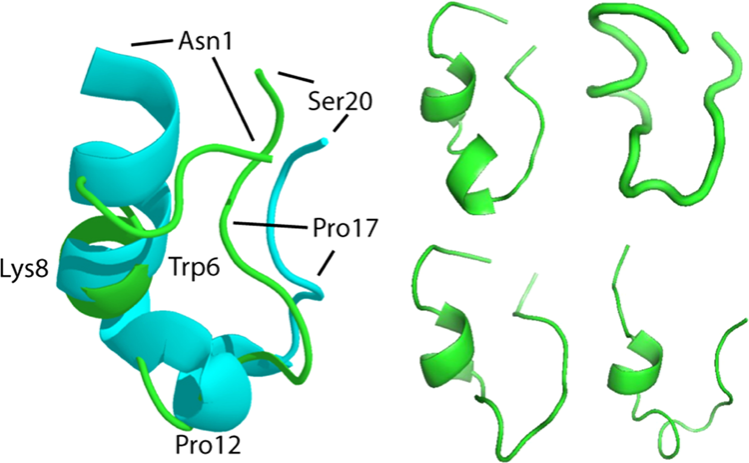
Conformations of Trp-cage in 28% HFIP-water at 298 K observed during simulations of up to 0.6 μs duration (green). The cyan structure is the conformation of the peptide at 283 K in water found by the work of Neidigh.^13^

**Figure 4:**
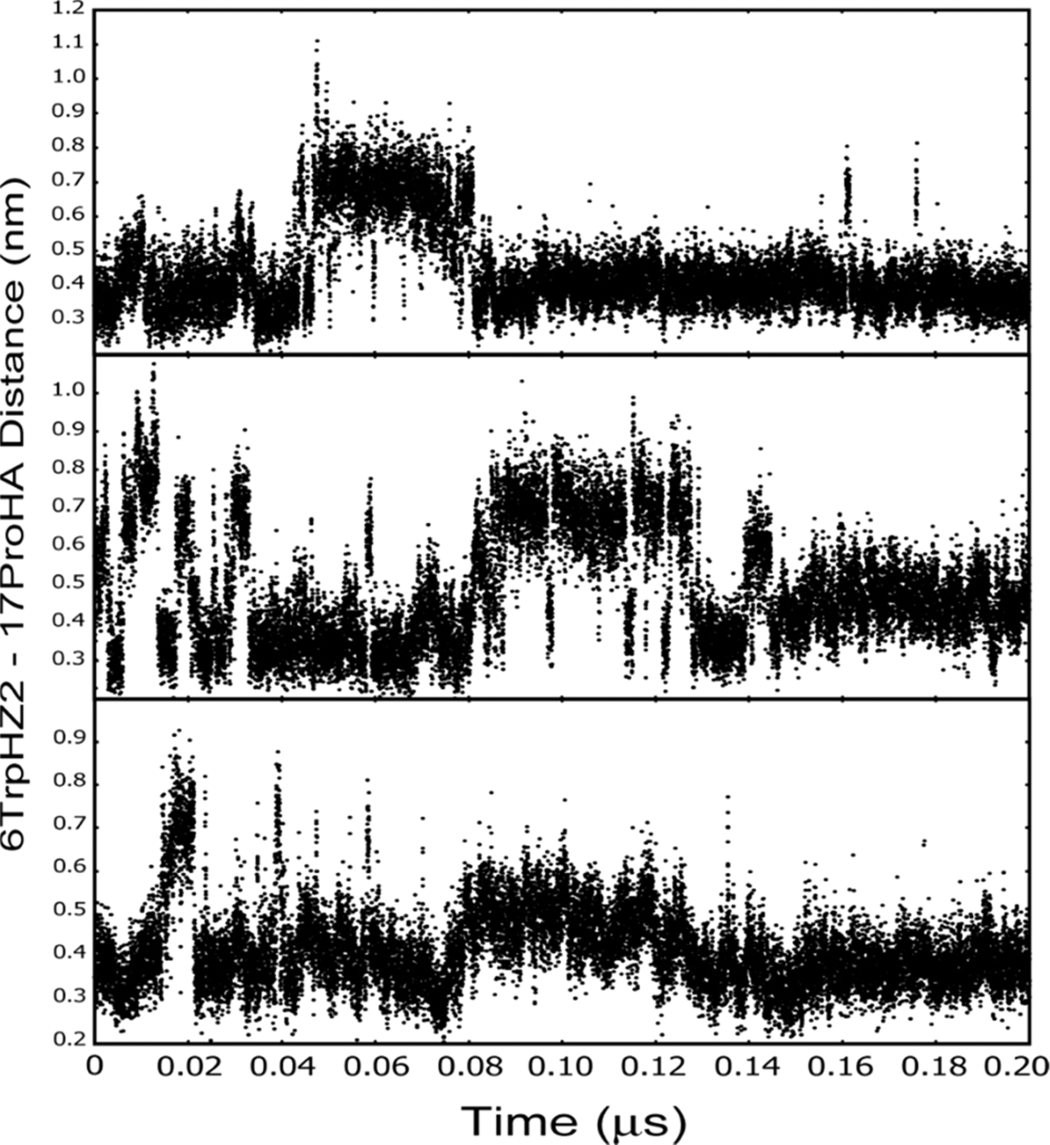
Variation of the 6TrpHZ2–17ProHA interhydrogen distance in simulations of Trp-cage in 28% HFIP-water at 298 K done with different atom velocities at the start of each simulation. The average distance found in all simulations done for this work was 0.44 nm. The intramolecular ^1^H–^1^H NOE arising from this interaction indicated an average hydrogen–hydrogen distance of 0.4 nm in pure water.^13^

Simulations done for the present work started with the Trp-cage molecule in the conformation (1L2Y) reported by Neidigh et al.^13^ Within 1 ps, the structure relaxed to hairpin structures in which some features of the initial structure were retained (Figure 3). The conformational mobility of the peptide during a simulation depended on the assigned initial atom velocities. As an example, Figure 4 shows the variation of the distance between hydrogens 6TrpHZ2 and 17ProHA in replicate simulations of the system, each started with a different set of initial atomic velocities. This distance ranged from 0.22 to 1.1 nm in different calculations; the averaged interhydrogen distance found in five independent simulations was 0.44 nm, in agreement with the interproton distance deduced from the experimental intramolecular NOE detected for this interaction at 283 K in water.^13^

Simulations at 298 K that used simulation boxes 5.25, 6.70, and 8.35 nm on a side, with the number of HFIP molecules present ranging from 228 to 1025, were examined in the present work (Supporting Information). Analysis of simulations of 0.1 μs duration showed that averaged conformation-defining proton–proton distances were close to distances indicated by proton intramolecular NOE experiments with the peptide in pure water.^13^ Simulations that ran longer (0.6 μs) showed some deviations from these.

### Solvent Distribution Near Trp-Cage

**Figure 5:**
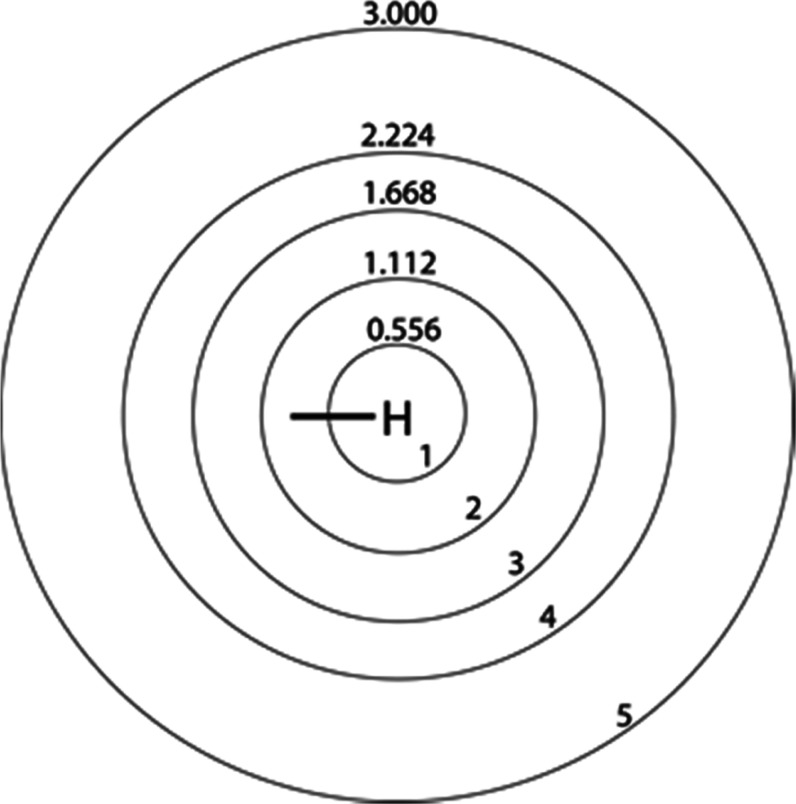
Shells of solvent centered on a hydrogen of Trp-cage. A portion of each solvent shell may be occupied by all or part of the solute. Sphere radii are spaced by 0.556 nm, the estimated diameter of a rapidly rotating HFIP molecule.^26^ Solvent molecules contained within 3.0 nm of a peptide hydrogen are used in computing peptide hydrogen–solvent fluorine cross-relaxation terms.

**Figure 6:**
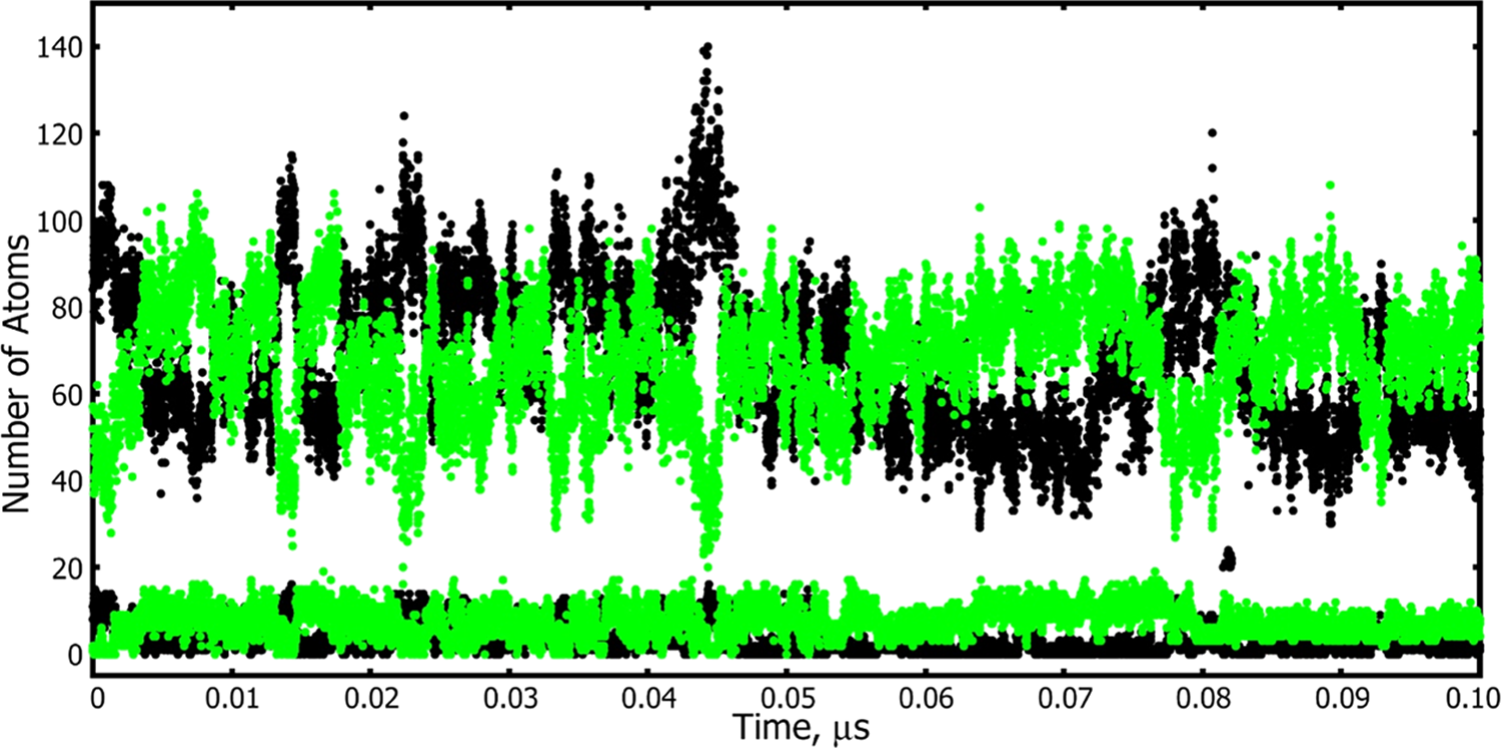
Changes in the composition of the solvent shell surrounding the 6TrpHE1 hydrogen of Trp-cage in 28% HFIP-water during a simulation at 298 K. The number of HFIP fluorine atoms is plotted with green symbols while the number of water oxygen atoms is plotted with black symbols. Composition data was collected every 10 ps. Data for solvent shell 1 is plotted at the bottom of the figure, while the composition of shell 1 + shell 2 is plotted above. The average number of fluorine atoms in shell 1 + shell 2 in this particular simulation was 67.6, while the average number of water oxygens was 67.9, giving a water oxygen-to-HFIP fluorine atom ratio of ∼1. In a sample of the solvent with no peptide present, this ratio is 2.54.

Solvent interactions with the hydrogens of Trp-cage in 28% HFIP-water were considered using the solvent shells defined in Figure 5. As an example, Figure 6 shows variations in the content of solvent shells 1 and 2 about hydrogen 6TrpHE1, found near the center of the Trp-cage structure. These solvent layers are highly dynamic, changing on a ps time scale. Ignoring the presence of Trp-cage, a homogeneous mixture of 7434 waters and 482 HFIP molecules contained in a 6.7 nm^3^ simulation box has a ratio of water molecules to HFIP fluorines of 2.57. Over the course of a 0.1 μs simulation, the ratio of water molecules to HFIP fluorines in solvent shells 1 and 2 around 6TrpHE1 ranged from a highly water-rich (∼7) to water-poor (∼0.3), with an average water-to-HFIP ratio of ∼1 reflecting a local average solvent composition about this peptide hydrogen that is richer in HFIP than is the case for the pure HFIP/water solvent mixture. Thus, in this simulation, the 6TrpHE1 hydrogen of the peptide is preferentially solvated by HFIP molecules.

The average occupancy of the solvent shells defined in Figure 5 about other hydrogens of Trp-cage was calculated for four independent simulations of 0.2 μs duration. Some results of these analyses are given in Table 2, with additional results given in the Supporting Information. These calculations indicated that solvent layer 1, immediately adjacent to peptide hydrogens of the N- and C-terminal regions of Trp-cage, as well as parts of the 6Trp, 9Asp, Ser13, and Ser14 residues, preferentially contains water molecules while this layer around the remaining peptide hydrogens is enriched in the fluoroalcohol. The second solvent layer adjacent to all hydrogens of the peptide (shell 2) is preferentially enriched in HFIP molecules. The third solvent layer (shell 3) is still enriched in the fluoroalchol, but less than shell 2. Shell 5 surrounding each peptide hydrogen of Trp-cage is water-rich (data not provided), having a water-to-HFIP ratio of 2.60 that is virtually invariant at each peptide hydrogen. Water molecules displaced from the inner solvent shells appear near the edges of the simulation box.

**Table 2:** Representative Calculated Average Occupancies of Solvent Shells^a^

peptide	HFIP	HFIP	HFIP	H_2_O	H_2_O	H_2_O	H_2_O/HFIP		
hydrogen	shell 1	shell 2	shell 3	shell 1	shell 2	shell 3	shell 1	shell 2	shell 3
1AspHA	1.54	42.64	148.89	10.98	101.76	290.45	7.1	2.4	2.0
6TrpH	0.94	50.35	163.80	3.65	64.95	302.35	3.9	1.3	1.8
6TrpHA	0.46	50.87	168.09	3.25	61.16	302.69	7.1	1.2	1.8
6TrpHD1	1.00	54.95	164.34	3.04	61.59	301.77	3.0	1.1	1.8
6TrpHE1	2.62	61.22	157.36	2.20	64.67	299.08	0.8	1.1	1.9
6TrpHZ2	7.26	61.77	151.90	2.09	66.91	299.21	0.3	1.1	2.0
6TrpHH2	11.20	60.38	147.65	2.79	69.16	298.23	0.2	1.1	2.0
6TrpHZ3	10.64	60.78	146.63	2.56	65.98	300.97	0.2	1.1	2.1
6TrpHE3	5.43	60.90	152.19	2.18	56.70	308.87	0.4	0.9	2.0
8LysH	3.17	51.87	156.76	1.67	72.80	298.53	0.5	1.4	1.9
12ProHA	5.83	62.36	154.89	3.90	72.78	296.10	0.7	1.2	1.9
17ProQG	9.25	56.04	141.80	8.32	86.54	295.97	0.9	1.5	2.1
20SerH	2.32	47.21	150.92	12.51	93.50	281.81	5.4	2.0	1.9

a Shell 1 is the solvent layer from 0 to 0.556 nm from the center of a peptide hydrogen atom. Shell 2 is the solvent layer from 0.556 to 1.112 nm, while Shell 3 includes the solvent layer from 1.112 to 1.657 nm. A rapidly rotating HFIP molecule is represented by a sphere 0.556 in diameter.^26^ The columns labeled HFIP provide the calculated average number of solvent fluorine atoms in a given shell. These atoms may or may not be on the same HFIP molecules. Mean deviations from the average varied but were of the order ±20% for Shell 1 and ±2% for Shells 2 and 3. A water-to-HFIP ratio greater than 2.56 corresponds to the local solvent mixture being enriched in water.

Comparison of the calculated contents of solvent shells around a given peptide hydrogen at 278 and 298 K indicate that HFIP contents of the shells are similar at the two temperatures when the difference in solvent composition (28% HFIP vs 30% HFIP) is taken into account. Solvent shells around the hydrogens attached to the aromatic ring of 6Trp appear to be an exception, with the contents of shells 1 and 2 in these instances being slightly richer in HFIP at 298 K than would be expected from the amounts of HFIP present in these shells at 278 K.

### Duration of Trp-Cage–Solvent Contacts

An alternate view of peptide hydrogen–solvent interactions in solvent shell 1 is obtained by enquiring about the number of solvent atom contacts with a peptide hydrogen of interest. For the purposes of this exercise, a hydrogen–fluorine contact was regarded as present when the hydrogen–fluorine distance was 0.5 nm or less. A peptide hydrogen–water contact was defined as having a water oxygen–peptide hydrogen distance of 0.4 nm or less. (These distances are arbitrary and correspond roughly to the radius of a solvent molecule plus a nonbonded contact distance of 0.2 nm.^26^) Recalling that snapshots of simulations were recorded every 10 ps, a contact was regarded as persistent when it was found within the indicated distance in subsequent snapshots. Thus, the resolution of a calculated contact duration is of the order ±10 ps. As anticipated, there was a linear relation between the average occupancy of shell 1 by HFIP and water and the number of solvent contacts as defined here (Supporting Information).

**Figure 7:**
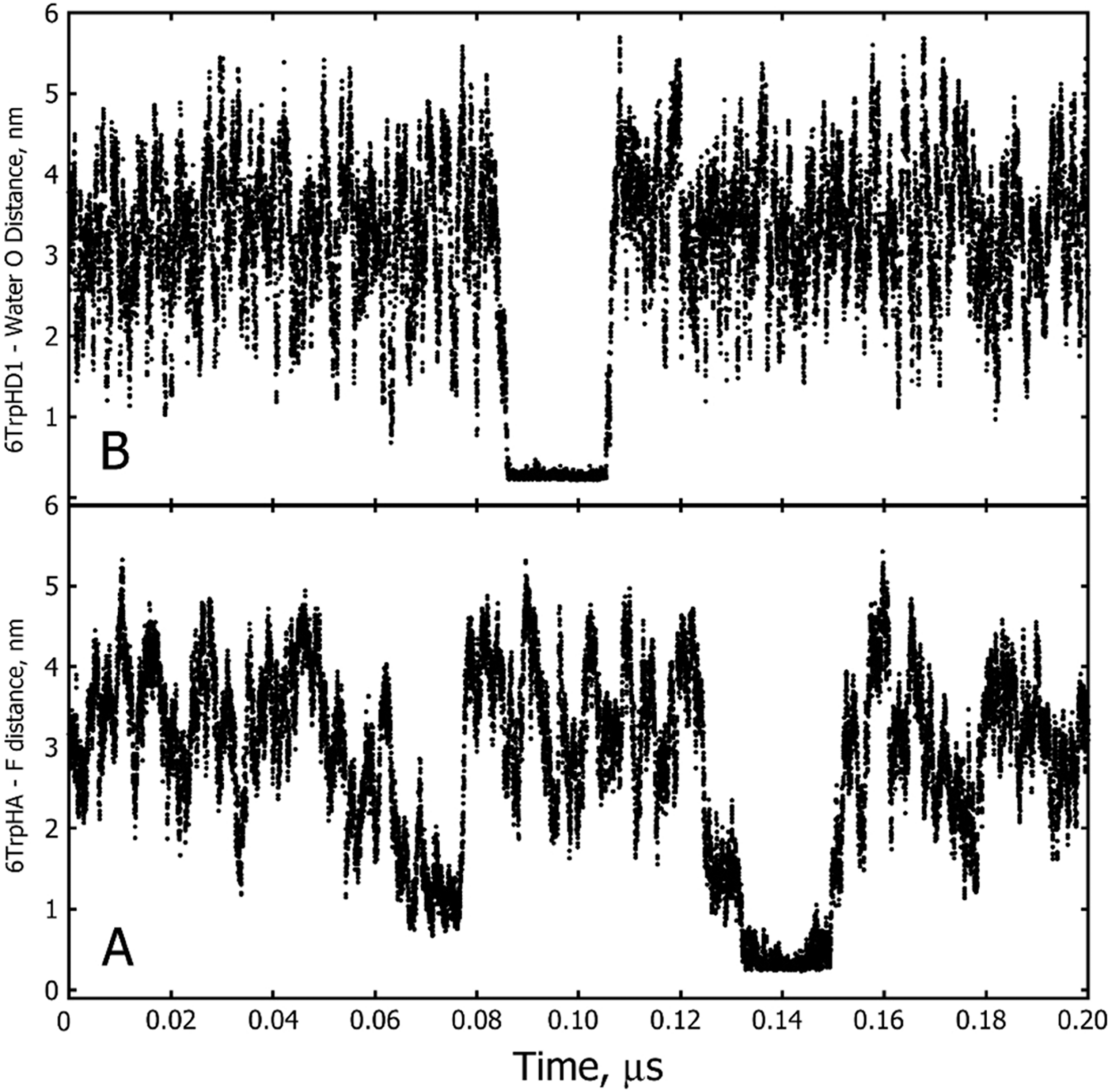
Persistent solvent interactions with Trp-cage in 28% HFIP-water at 298 K. (A) Distance between a fluorine atom of an HFIP molecule and 6TrpHA of Trp-cage. The average H–F distance for the interaction near 0.14 μs was 0.37 ± 0.09 nm. (B) Distance between the oxygen atom of a water molecule and 6TrpHD1. The average H–O distance for the interaction near 0.10 μs was 0.27 ± 0.02 nm.

Table 3 shows some results of this analysis. It was found that most contacts with HFIP fluorine atoms and water molecules were broken in 20 ps or less. However, a few contacts evolved into interactions that lasted on the order of 0.01 ns or longer. Longer-lasting solvent interactions tended to be found in the vicinity of hydrogens of 6Trp, 12Pro, and 16Arg. While very long solvent interactions were not observed in every simulation, Figure 7 shows that peptide hydrogen–solvent fluorine interactions lasting ∼20 ns were occasionally observed.

**Table 3:** Solvent Contacts with Selected Trp-Cage Hydrogens in 28% HFIP-Water

Trp-cage hydrogen	average *F* contacts per ns	contacts longer than 60 ps (%)	average *F* contact duration^a^	average H_2_O contacts per ns	contacts longer than 60 ps (%)	average H_2_O contact duration^a^
1AsnHD22	298	2.9	115	489	2.6	123
6TrpH	51	1.8	120	151	1.6	568
6TrpHA	42	3.6	171	72	3.3	145
6TrpHD1	61	8.0	128	118	2.8	226
6TrpHE1	146	2.0	109	79	2.5	225
6TrpHH2	743	2.5	96	79	0.7	240
6TrpHZ2	435	3.2	110	71	2.6	134
6TrpHZ3	730	2.9	99	78	0.7	83
6TrpHE3	339	2.4	106	42	1.0	90
8LysH	174	1.6	93	8	0.9	116
12ProHA	361	3.0	114	69	3.9	202
17ProHD2	430	2.5	109	145	1.3	144
20SerH	129	2.2	118	246	2.4	158

a Average duration of contacts longer than 60 ps.

### Solvent Diffusion Near Trp-Cage

Intermolecular nuclear dipole–dipole relaxation depends critically on the mutual diffusion of the interacting partners.^28^ Experimentation and MD simulations indicate that diffusion of solvent water is slowed near the surface of proteins.^46−51^ Considerations related to the structural, chemical, and dynamical heterogeneity of the surface are responsible for the slowing.^52−54^ The dynamics of water on the surface of a protein likely have implications for biological activity.^55^

**Figure 8:**
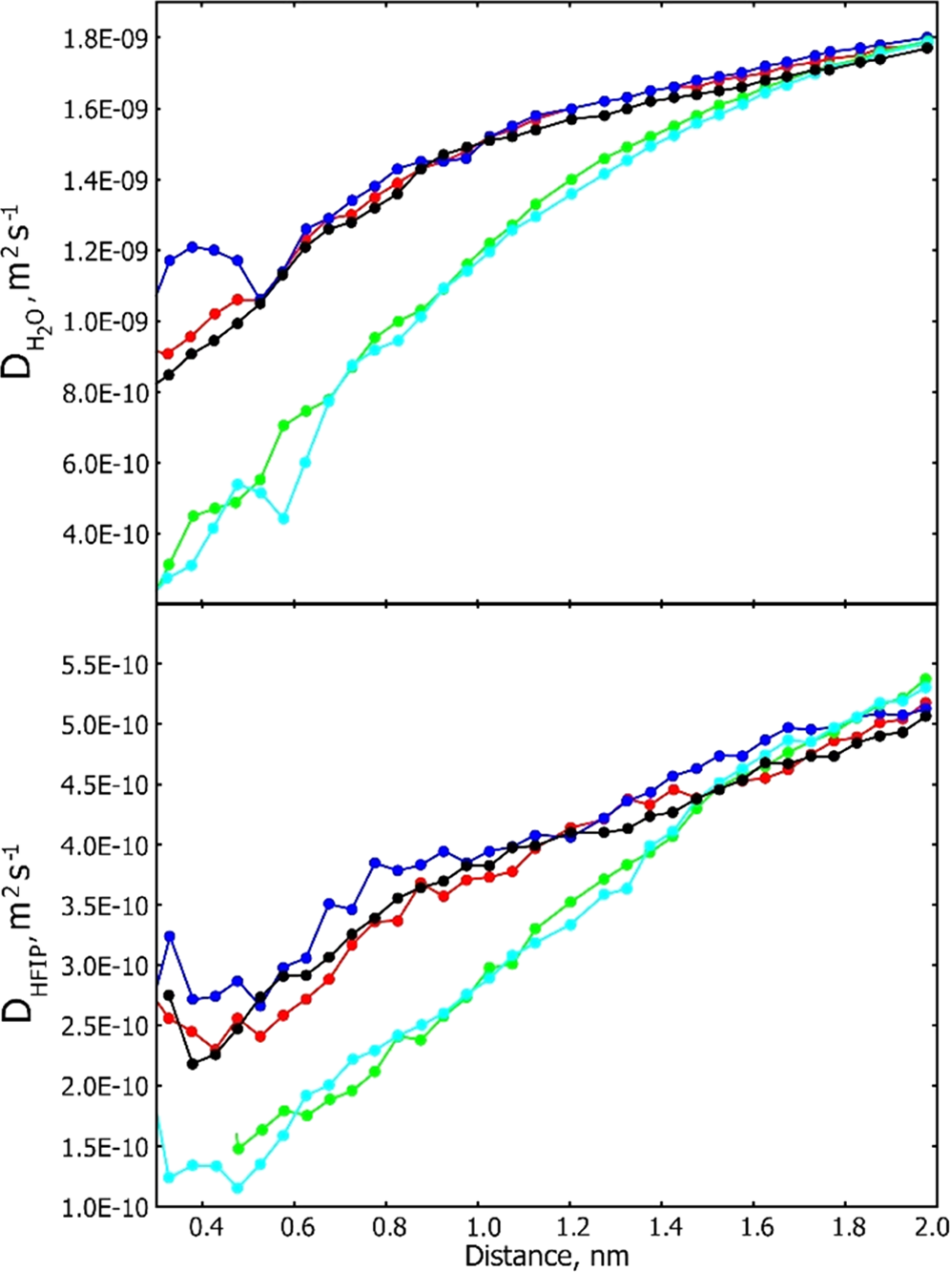
Calculated translational diffusion coefficients as a function of distance from a Trp-cage hydrogen atom in 28% HFIP-water at 298 K. Red, blue, and black symbols represent data for the 1AspHB3, 3TyrH, and 20SerH protons of Trp-cage, respectively, while green and cyan symbols represent data for the 6TrpH and 6TrpHD1 protons of the peptide, respectively. The calculated translational diffusion coefficients for HFIP and water in the bulk solvent at 298 K are 4.8 × 10^–10^ and 17. × 10^–10^ m^2^ s^–1^ (Table 1).

The approach of Pettit and co-workers was used to explore diffusion of HFIP and water near the surface of Trp-cage at 298 K.^56^ Some typical results are shown in Figure 8. The calculations suggest a modest retardation (∼50%) of translational diffusion of the fluoroalcohol near most Trp-cage hydrogens. Diffusion of water near the same peptide atoms seems to be slowed by about the same amount. However, diffusion of the solvent components near the tryptophan residue of Trp-cage is more strongly influenced by the peptide, with diffusion near the peptide being reduced by a factor of ∼4 compared to diffusion in the rest of the sample. Reduction of translational diffusion of either solvent component is consistent with the observation that some solvent molecules can persist for relatively long times in the solvent shells of peptide hydrogens (Table 3).

### Trp-Cage Hydrogen-HFIP Fluorine Cross-Relaxation

Experimental peptide hydrogen–solvent fluorine cross-relaxation parameters (Σ_HF_) for most hydrogens of Trp-cage in the present system were generally small and of either sign (Figure 1). Cross-relaxation parameters calculated from simulations of Trp-cage in 28% HFIP-water at 298 K are given in the Supporting Information. In Table 4, some of the calculated Σ_HF_ parameters are compared to experimental results.

**Table 4:** Observed and Calculated Cross-Relaxation Terms (Σ_HF_)^a^

hydrogen	shift, ppm	Σ_HF_, ×10^3^ s^–1^ (obs)	Σ_HF_, ×10^3^ s^–1^ (calc)
6TrpHE1	9.862	–5	–7 ± 1
8LysH	9.099	–3	–4 ± 1
11GlyH	8.687	–2	–3 ± 1
7LeuH	8.416	–3	–4 ± 1
14SerH	8.197	∼0	–2 ± 1
6TrpH	8.178	∼0	–6 ± 3
16ArgH	8.163	–2	–7 ± 1
3TyrH	8.131	∼0	–1 ± 1
5GlnH + 9AspH	8.080 + 7.990	–3	–5 ± 3^a^
4IleH	7.829	∼0	–7 ± 5
2LeuH + 10GlyH + 15GlyH	7.777 + 7.705 +7.744	∼0^a^	–2 ± 1
13SerH	7.694	∼0	0 ± 1
6TrpHE3 + 5GlnHE21	7.555 + 7.549	∼0	–1 ± 2^a^
1AsnHD21 + 20SerH	7.296 + 7.290	∼0	0 ± 1^a^
6TrpHZ3 + 6TrpHZ2	7.221 + 7.205	1	1 ± 3^a^
6TrpHH2	7.138	∼0	7 ± 1
6TrpHD1 + 16ArgHE	7.107 + 7.090	–7	–12 ± 8^a^
3TyrQE	6.852	4	5 ± 1
5GlnHE22	6.566	∼0	–2 ± 4
1AsnHD22	6.204	6	1 ± 2
8LysHA	3.948	2	3 ± 1
16ArgHD2 + 6TrpHB3	3.338 + 3.327	–9	–2 ± 2^a^
9AspHB2	2.974	-4	–4 ± 4
18ProHA	2.760	–6	–4 ± 1
12ProHB3	2.542	–6	3 ± 3
5GlnQG	2.441	–1	–5 ± 2
5GlnQB	2.281	–11	–5 ± 2
18ProHB2	1.351	4	0 ± 1
4IleQD1 + 7LeuQD1 + 2LeuQD1	1.016 + 1.005 + 0.998	4	3 ± 3^a^
Ile4QG2 + 7LeuQD2 + 2LeuQD2 + 11Gly HA2	0.968 + 0.958 + 0.954 + 0.942	2	1 ± 1^a^
18ProHB3	0.368	∼0	–2 ± 2

a The average calculated Σ_HF_ is shown for overlapped signals.

It was found that a major share of a calculated Σ_HF_ arises from fluorine-peptide hydrogen contacts that take place in solvent shell 1 (Figure 5) with interactions in solvent shell 2 also being significant in defining the sign and magnitude of Σ_HF_. The contribution of HFIP–peptide interactions to Σ_HF_ from HFIP molecules in solvent shell 3 is nearly constant for all Trp-cage hydrogens at about −0.6 × 10^–4^ s^–1^. The contribution from solvent shell 4 is −0.4 × 10^–4^ s^–1^. The contributions of shells 1 and 2 to Σ_HF_ about a peptide hydrogen is 1–2 orders of magnitude larger than the contributions from the remainder of the sample. Thus, the variation of calculated Σ_HF_ parameters is largely the result of the details of the structure and dynamics of peptide–solvent interactions in the immediate environment of the peptide.

Simulations of this system at 278 K, with some exceptions, predicted the correct sign of Σ_HF_ and usually the correct magnitude of the cross-relaxation parameter Σ_HF_. However, for many hydrogens of Trp-cage at 298 K the uncertainties in Σ_HF_, as suggested by the mean deviation from the mean, are large. We note that generally the results with the largest uncertainties arise in situations where there are indications of long-lasting solvent interactions with the peptide. When they are present, these interactions have the effect of making the calculated Σ_HF_ for a simulation algebraically smaller than anticipated and thereby widening the uncertainty of Σ_HF_.

Some of the signals in the ^1^H spectrum of Trp-cage are overlapped at 298 K (Table 4). It was assumed that the contributions of all overlapped signals to an observed NOE enhancement are equal for each component when calculating the enhancement.

## Discussion

Force field parameters used in the present work were the same as those used for an earlier study of the system at 278 K.^22^ These parameters led to predictions of the translational diffusion coefficients of the peptide and HFIP components of the system at both 278 and 298 K that agreed with experiment; the diffusion coefficient of water in both cases was overestimated. This is often the case in simulations of alcohol–water mixtures^57−59^ and reflects the difficulty in finding a parameter set that is equally reliable for simulations of pure water, alcohol, peptide components, and their combination in mixtures.

Comparison of the contents of solvent shells 1 through 3 at 278 and 298 K shows that, after taking into account the difference in the bulk solvent compositions (30% vs 28% HFIP), most solvent shells about each Trp-cage hydrogen are populated to essentially equivalent extents by HFIP and water at the two temperatures. Exceptions were found at the 6Trp residue. It was calculated that the 6TrpH, 6TrpHA, 6TrpHB2, 6TrpHB3, and 6TrpHD1 hydrogens are less solvated by HFIP in solvent shell 1 at 298 K than would be expected from the contents of shell 1 at 278 K. The remaining 6Trp hydrogens are significantly more solvated by HFIP at 298 K than would be expected from results obtained at 278 K. An implication is that the free energy change associated with HFIP interactions at the former 6Trp hydrogens is larger than that characteristic of interactions at the remaining tryptophan hydrogens, making these interactions more sensitive to sample temperature.

Solvent layer 1 at the N- and C-terminals of Trp-cage is water-rich at 298 K as is solvent layer 1 around the hydrogen atoms at the junction of the 5Gln and 6Trp residues. Solvent layer 1 around the remaining peptide hydrogens is rich in HFIP. All peptide backbone hydrogens are preferentially solvated by HFIP in shells 2 and 3. Thus, overall, Trp-cage is “coated” with HFIP. It has long been known that fluoroalcohols tend to aggregate on the surface of peptides,^8,60,61^ and our results are consistent with many previous studies of small peptides in fluoroalcohol–water systems.

Calculations show that, in most cases, the major portion of a cross-relaxation parameter for the interaction of HFIP with a hydrogen of Trp-cage at 298 K is the result of interactions of fluorines atoms within ∼1 nm of the hydrogen (solvent shells 1 and 2). The computations of the cross-relaxation parameters (∑_HF_) done here for the system at 298 K used the same methodology as was used for previous work with the system at 278 K.^22^ While several ∑_HF_ values at 298 K are reasonably predicted by the calculations done (Table 4), overall the agreement between observed and calculated ∑_HF_ values is poorer at the higher temperature. As indicated earlier, at 298 K, many of the intermolecular ^1^H{^19^F} NOEs found for the present system are relatively small in magnitude and of either algebraic sign. Computationally, a ∑_HF_ value is the result of the difference between two quantities that are numerically closer in magnitude at 298 K than they are at 278 K. Obtaining good predictions of solvent–solute cross-relaxation terms at 298 K is thus more sensitive to the reliability of the approximations and assumptions made in calculating them.

Complicating any attempt to understand the reasons for the lack of agreement between observed and calculated ∑_HF_ is the observation that some peptide–solvent interactions can persist in the simulations for times up to several ns. These long-lived interactions were not detected in all simulations done for this work, and it may be that simulations of longer duration than those used here may be required to better account for the influence of these interactions on ∑_HF_.

## Conclusions

An MD force field previously used for simulations of Trp-cage in a mixture of water and HFIP gave good predictions of the diffusion coefficients for the peptide and fluoroalcohol and led to reasonable predictions of the aggregation of HFIP in the system at 278 K. Using the same force field at 298 K similarly gave good predictions of these features of the Trp-cage/water/HFIP system at 298 K in simulations lasting up to 0.6 μs. Simulations indicated that a few peptide hydrogens in addition to the N- and C-termini have water molecules in the solvent layer immediately adjacent to peptide. Beyond this first layer, all peptide hydrogens are preferentially solvated by interactions with HFIP molecules. Peptide hydrogen magnetic dipolar interactions with fluorine atoms of the solvent mixture are dominated by interactions with the fluoroalcohol within the first two solvent layers around the peptide hydrogen. At 298 K, many experimental hydrogen–fluorine cross-relaxation parameters (∑_HF_) for these interactions are small and obscured by spectral noise. There is generally only modest agreement between ∑_HF_ calculated from simulations and experimental data.

There are suggestions from this work that HFIP interactions with the tryptophan residue of Trp-cage and its vicinity in the peptide may be stronger and longer-lasting than interactions with other residues of the peptide (Table 3 and Figures 7 and 8). Interestingly, the experimental sign and magnitude of ∑_HF_ calculated for interactions of some hydrogen atoms of 6Trp agree with the predictions of the simulations.

## Supporting Information Available

Force field parameters used, mean intramolecular ^1^H–^1^H distances in the peptide in simulations, calculated occupancy by solvent molecules of solvent shells about peptide hydrogens, calculated solvent atoms contacts with the peptide, and calculated ∑_HF_ parameters for interaction of HFIP with Trp-cage (PDF)
