# Supplementary material for: Examination of Solvent Interactions with Trp-Cage in 1,1,1,3,3,3-Hexafluoro-2-propanol-water at 298 K through MD Simulations and Intermolecular Nuclear Overhauser Effects
Source: J Phys Chem B. 2023 May 30;127(22):5062–71. doi: 10.1021/acs.jpcb.3c01029 (PMC10258800; doi:10.1021/acs.jpcb.3c01029)
Supplement: Supplementary file 1 — jp3c01029_si_001.pdf [file jp3c01029_si_001.pdf]

Supporting Information

Examination of Solvent Interactions with Trp-Cage in 1,1,1,3,3,3-Hexafluoro-2-propanol-Water at 298 K through MD Simulations and Intermolecular Nuclear Overhauser Effects

J. T. Gerig

Department of Chemistry & Biochemistry

University of California, Santa Barbara

Santa Barbara, CA 93106 U.S.A.

I. Parameters for Simulations

Parameters for non-bonded interactions not mentioned here or in the main text were the standard values for the AMBER99SB-ILDN force field.[1-2]

Symbol	Mass	Sigma, nm	Epsilon ,kJmol-1	Use
F	19.00	0.3151	0.4170	HFIP atom
D1	2.014	0.2471	0.6589	HFIP tertiary hydrogen
D0	2.014	0.	0.	HFIP hydroxl hydrogen
HW_tip5p	1.008	0.	0.	Water hydrogen
OW_tip5p	16.00	0.3097	0.7448	Water oxygen
F - H		0.2321	0.1655	
F - H1		0.3092	0.1655	
F - HP		0.2811	0.1655	
F - HC		0.3191	0.1655	
F - HA		0.3163	0.1618	
F – H2		0.2994	0.1655	
F - H3		0.2896	0.1655	
F - HO		0.1733	0.	
F - HW		0.1733	0.	
F - D1		0.3092	0.1655	
F - D0		0.1733	0.	

The combination rule for σ non-bonded parameters was the arithmetic mean ($\sigma_{ij} = \frac{1}{2}\left(\sigma_{ii} + \sigma_{jj}\right)$as used in the AMBER force fields, with the result multiplied by 1.10. The combination rule for ε_{ij} non-bonded parameters was the geometric mean ($\varepsilon_{ij} = \left(\varepsilon_{ii}\varepsilon_{jj}\right)^{1/2}$).

II. Tryptophan Nomenclature

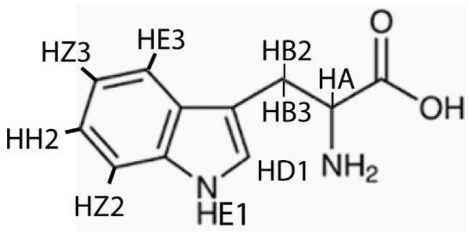

III. Distances and Intramolecular $^{1}H\{^{1}H\}$ NOEs in Trp-Cage Simulations

Spin 1-Spin 2	r_{sim}, *nm*[a]	r_{sim}, *nm*[b]	r_{sim}, *nm*[c]	r_{exp}, *nm*[d]
Box edge, nm	*5.25*	*6.70*	*8.35*	
2LeuH – 4IleH	0.47	0.63	0.44	0.40
2LeuH – 5GlnH	0.54	0.51	0.49	0.30
3TyrHA – 6TrpH	0.33	0.58	0.32	0.35
3TyrHA – 6TrpHE3	0.38	0.86	0.38	0.35
3TyrHA – 19ProHB2	0.54	0.81	0.52	0.40
3TyrHA – 19ProHD2	0.41	0.90	0.38	0.35
4IleH – 5GlnH	0.27	0.26	0.27	0.30
4IleHA – 5GlnH	0.36	0.32	0.36	0.35
4IleHB – 5GlnH	0.31	0.42	0.30	0.30*
4IleHA – 7LeuH	0.33	0.45	0.33	0.30
5GlnH – 7LeuH	0.41	0.51	0.43	0.40*
5GlnHA – 8LysH	0.36	0.37	0.33	0.30
6TrpHA – 6TrpHD1	0.30	0.26	0.29	0.30*
6TrpHB2 – 6TrpHD1	0.38	0.38	0.38	0.30
6TrpH – 6TrpHE3	0.46	0.49	0.46	0.40*
6TrpH – 7LeuH	0.27	0.29	0.27	0.30*
6TrpHE3 – 7LeuHG	0.29	0.42	0.27	0.25*
6TrpHZ3 – 7LeuHG	0.41	0.56	0.40	0.40*
6TrpHZ2 – 12ProHA	0.30	0.32	0.29	0.25*
6TrpHH2 – 12ProHA	0.41	0.45	0.41	0.40*
6TrpHE1 – 16ArgH	0.36	0.40	0.35	0.40
6TrpHE1 – 17ProHA	0.38	0.38	0.37	0.35*
6TrpHZ2 – 17ProHA	0.38	0.44	0.38	0.40*
6TrpHZ2 – 18ProHD1	0.45	0.37	0.44	0.35
6TrpHZ2 – 18ProHD2	0.29	0.53	0.28	0.40
6TrpHZ2 – 18ProHB1	0.55	0.64	0.54	0.40*
6TrpHE1 – 18ProHA	0.36	0.37	0.36	0.35*
7LeuH – 9AspH	0.42	0.40	0.42	0.40
7LeuH – 9AspH	0.42	0.42	0.42	0.40
7LeuHA – 10GlyH	0.36	0.34	0.35	0.40*
7LeuHA – 11GlyH	0.27	0.51	0.26	0.25*
8LysHA – 9AspH	0.35	0.35	0.35	0.35
8LysH – 9AspH	0.28	0.26	0.28	0.25*
8LysH – 10GlyH	0.42	0.45	0.41	0.25
8LysH – 11GlyH	0.49	0.54	0.48	0.40
10GlyH – 11GlyH	0.24	0.48	0.23	0.25*
12ProHA – 13SerH	0.35	0.35	0.35	0.35
12ProHA – 14SerH	0.38	0.40	0.37	0.40*
12ProHA – 15GlyH	0.44	0.41	0.45	0.40
13SerHA – 14SerH	0.33	0.34	0.33	0.35
13SerHA – 15GlyH	0.37	0.38	0.36	0.35*
17ProHA – 19ProHD1	0.68	0.68	0.68	0.25
17ProHA – 19ProHD2	0.60	0.62	0.62	0.30

18ProHA – 19ProHD1	0.25	0.25	0.25	0.25*
19ProHB2 – 20SerH	0.36	0.34	0.36	0.35

[a] The system simulated involved a Trp-cage molecule with 228 HFIP molecules, 3516 water molecules and a chloride ion. Average hydrogen-hydrogen distances found in 5 simulations (0.1μs duration) of Trp-cage in 28% hexafluoroisopropanol-water at 298 K.

[b] The system simulated involved a Trp-cage molecule with 482 HFIP molecules, 7434 water molecules and a chloride ion. Average hydrogen-hydrogen distances found in 10 simulations of up to 0.6 μs duration of Trp-cage in 28% hexafluoroisopropanol-water at 298 K.

[c] The system simulated involved a Trp-cage molecule with 1025 HFIP molecules, 14000 water molecules and a chloride ion. Average hydrogen-hydrogen distances found in 10 simulations (0.1μs duration) of Trp-cage in 30% hexafluoroisopropanol-water at 298 K.

[d] Experimental intramolecular H-H distances in water at 283K reported by Neidigh, *et al*.[3] Asterisks indicate that the corresponding ^{1}H-^{1}H intramolecular NOEs were observed in studies of Trp-cage in 30% hexafluoroisopropanol-water at 298 K.[4]

Table IV. Calculated Occupancy of Solvent Shells[a]

Peptide Hydrogen	HFIP Shell 1	HFIP Shell 1+2	HFIP Shell 1+2+3	H2O Shell 1	H2O Shell 1+2	H2O Shell 1+2+3	Ratio[b] Shell 1	Ratio[b] Shell 1+2	Ratio[b] Shell 1+2+3
1AspHA	1.54	44.18	193.07	10.98	112.74	403.19	7.1	2.6	2.1
1Asp HB2	2.88	45.47	196.65	11.98	109.76	398.83	4.2	2.4	2.0
1AsnHH21	3.84	46.07	197.35	12.80	82.75	399.57	3.3	1.8	2.0
1AsnHH22	4.37	46.83	195.80	12.82	108.29	399.46	2.9	2.3	2.0
2LeuH	1.70	44.76	195.58	7.94	105.62	397.73	4.7	2.4	2.0
2LeuQD1	4.81	48.77	187.13	12.17	115.67	415.69	2.5	2.4	2.2
2LeuQD2	3.91	48.99	186.62	12.91	118.93	420.28	3.3	2.4	2.3
3TyrH	3.58	51.96	194.73	9.29	105.15	404.01	2.6	2.0	2.1
3TyrHB2	5.26	55.21	196.69	10.39	106.25	405.57	2.0	1.9	2.1
3TyrHB3	4.70	56.33	196.68	9.92	103.94	405.02	2.1	1.8	2.1
3TyrQD	5.84	59.16	200.14	9.59	101.05	403.01	1.6	1.7	2.0
3TyrQE	6.84	63.60	202.75	10.49	104.04	400.35	1.5	1.5	2.0
4LeuH	2.19	56.28	207.58	4.98	82.41	382.07	2.3	1.5	1.8
4LeuQG2	6.77	64.80	205.66	4.92	83.14	384.12	0.7	1.2	1.9
4LeuQD1	7.10	65.20	211.72	4.73	72.23	374.43	0.7	1.1	1.8
5GlnH	1.71	51.66	205.74	4.69	82.38	379.12	2.7	1.6	1.8
5GlnHH21	4.19	43.41	197.08	10.24	101.52	390.11	2.4	2.3	2.0
5GlnHH22	3.80	44.07	192.82	11.21	107.24	396.51	2.9	2.4	2.1
5GlnQB	1.96	44.68	205.02	5.53	87.99	380.13	2.8	2.0	1.9
5GlnQG	3.43	43.00	203.39	7.37	91.99	381.99	2.1	2.1	1.9
6TrpH	0.94	51.29	215.09	3.65	68.60	370.95	3.9	1.3	1.7
6TrpHA	0.70	51.33	219.42	3.25	64.41	367.10	4.6	1.3	1.7
6TrpHB2	1.76	54.55	218.79	4.19	62.12	367.48	2.4	1.1	1.7
6TrpHB3	1.87	59.68	218.78	3.00	58.73	367.49	1.6	1.0	1.7
6TrpHD1	1.00	55.95	220.29	3.04	64.63	366.40	3.0	1.2	1.7
6TrpHE1	2.62	63.84	221.20	2.20	66.87	365.95	0.8	1.0	1.7
6TrpHZ2	7.26	69.03	220.93	2.09	69.00	368.20	0.3	1.0	1.7
6TrpHH2	11.20	71.58	219.23	2.79	71.94	370.17	0.2	1.0	1.7
6TrpHZ3	10.64	71.42	218.05	2.56	68.54	369.51	0.2	1.0	1.7
6TrpHE3	5.43	66.33	218.52	2.18	58.88	367.75	0.4	0.9	1.7
7LeuH	1.23	58.49	215.81	1.32	63.15	370.45	1.1	1.1	1.7
7LeuHA	3.02	62.51	217.92	1.98	65.74	368.91	0.7	1.1	1.7
7LeuHB3	7.80	63.66	211.89	2.84	73.63	374.54	0.4	1.2	1.8
7LeuQD1	10.24	67.67	211.30	3.40	75.57	376.67	0.3	1.1	1.8
7LeuQD2	8.94	68.77	214.72	2.60	70.08	372.67	0.3	1.0	1.7
8LysH	3.17	55.04	211.80	1.67	74.46	373.99	0.5	1.4	1.8
8LysHA	5.94	55.13	206.35	6.89	89.21	380.80	1.2	1.6	1.8
8LysQD	5.66	52.70	196.07	10.23	99.48	392.82	1.8	1.9	2.0
8LysQE	6.57	55.34	194.43	11.32	103.74	399.18	1.7	1.9	2.1
9AspH	1.19	48.59	214.42	3.59	76.40	371.36	3.0	1.6	1.7
9AspHB2	1.64	46.57	216.87	6.31	79.04	369.76	3.8	1.7	1.7
10GlyH	2.08	53.45	216.75	5.01	77.95	370.33	2.4	1.5	1.7

10GlyHA2	4.19	56.41	216.85	7.47	84.88	373.52	1.8	1.5	1.7
11GlyH	0.96	59.23	220.60	2.14	69.10	366.36	2.2	1.2	1.7
12ProHD2	5.61	69.28	220.76	3.44	74.12	372.42	0.6	1.1	1.7
12ProHB2	9.50	71.08	221.10	5.66	81.91	378.94	0.6	1.2	1.7
12ProHA	5.83	68.19	223.08	3.90	76.68	372.79	0.7	1.1	1.7
13SerH	3.01	64.36	222.97	5.71	82.79	375.71	1.9	1.3	1.7
13SerHA	4.90	63.63	220.66	10.41	97.29	387.54	2.1	1.5	1.8
13SerHB2	3.07	60.33	218.38	10.03	96.72	386.43	3.3	1.6	1.8
13SerHB3	3.61	60.22	216.12	11.98	103.34	391.95	3.3	1.7	1.8
14SerH	1.49	60.67	223.30	4.58	84.58	374.41	3.1	1.4	1.7
14SerHB2	1.87	54.71	219.18	6.45	88.45	375.82	3.4	1.6	1.7
15GlyH	4.10	62.62	221.77	5.57	88.57	379.80	1.4	1.4	1.7
15GlyHA2	7.51	64.68	218.31	8.12	93.13	387.52	1.1	1.4	1.8
16ArgH	3.11	59.84	217.02	3.90	83.23	375.29	1.3	1.4	1.7
16ArgHD2	4.79	56.13	212.71	8.83	92.87	382.94	1.8	1.7	1.8
16ArgHD3	4.99	54.57	210.44	9.28	95.23	384.81	1.9	1.7	1.8
16ArgHE	3.91	50.09	212.58	9.08	89.78	378.41	2.3	1.8	1.8
17ProHD2	6.81	62.83	209.28	7.32	90.94	389.02	1.1	1.4	1.9
17ProHB2	7.13	64.13	188.72	7.50	89.95	385.96	1.1	1.4	2.0
17ProHB3	9.93	66.83	209.52	6.43	90.13	386.02	0.6	1.3	1.8
17ProQG	9.25	65.29	207.09	8.32	94.86	390.83	0.9	1.5	1.9
18ProHD2	8.13	66.29	216.81	3.50	75.54	372.82	0.4	1.1	1.7
18ProHD3	8.15	65.49	213.16	5.68	83.24	377.13	0.7	1.3	1.8
18ProHB2	4.77	59.10	217.32	4.64	71.09	369.40	1.0	1.2	1.7
18ProHA	1.36	56.93	218.33	4.08	68.41	368.26	3.0	1.2	1.7
19ProHA	2.51	51.54	207.25	10.35	93.21	379.06	4.1	1.8	1.8
20SerH	2.32	49.53	200.45	12.512	106.01	387.822	5.4	2.1	1.9

[a] The columns labeled HFIP provide the calculated average number of solvent fluorine atoms in the indicated shell(s). These will not correspond to the number of intact HFIP molecules present. Results from 4 or more simulations of 0.2 μs duration were averaged. Mean deviations from the average varied but are of the order of ± 20% for Shell 1 and ± 2% for Shells 2 and 3. A water-to-HFIP ratio greater than 2.56 corresponds to the local solvent mixture about a peptide hydrogen being enriched in water.

[b] Ratio of water molecules to fluorine atoms.

Table V. Solvent Contacts with Trp-cage in 28% HFIP-Water

	Average F contacts per ns	***Contacts longer than 60 ps (%)***	***Average F contact duration***[a]	***Average H2O contacts per ns***	***Contacts longer than 60 ps (%)***	***Average H_2O contact duration***[a]
1AsnHD22	298	2.9	115	489	2.6	123
2LeuH	82	1.3	96	133	2.6	133
2LeuQD1	318	2.1	95	352	0.8	86
3TyrH	226	2.7	109	258	2.6	140
3TyrHB2	351	2.8	108	337	2.2	114
3TyrHB3	281	1.9	98	334	2.4	114
3TyrQD	387	5.4	100	288	2.6	98
3TyrQE	436	5.8	96	340	3.3	149
4IleH	125	1.4	108	189	3.3	216
4IleQD1	470	3.9	95	105	0.6	97
5GlnH	91	2.9	119	173	1.6	451
5GlnQB	103	3.2	95	162	3.2	236
5GlnHE21	290	3	114	389	3	134
6TrpH	51	1.8	120	151	1.6	568
6TrpHA	42	3.6	171	72	3.3	145
6TrpHB2	110	3	119	189	2.3	323
6TrpHD1	61	8	128	118	2.8	226
6TrpHE1	146	2	109	79	2.5	225
6TrpHH2	743	2.5	96	79	0.7	240
6TrpHZ2	435	3.2	110	71	2.6	134
6TrpHZ3	730	2.9	99	78	0.7	83
6TrpHE3	339	2.4	106	42	1	90
7LeuH	40	2.8	89	4	1.1	135
7LeuHA	161	2.5	107	23	1	98
7LeuQD1	677	4.6	93	92	0.5	104
8LysH	174	1.6	93	8	0.9	116
8LysHA	372	2.9	122	185	1.7	97
8LysQD	337	6.6	102	292	3.4	102
9AspH	61	2.2	100	21	0.6	95
10GlyH	108	1.4	95	73	0.9	98
10GlyHA2	287	3.4	110	281	2.2	102
11GlyH	47	1.3	94	27	2.3	186
12ProHA	361	3	114	69	3.9	202
13SerH	147	1.4	95	68	2.7	119
13SerHA	293	2.6	101	220	1.8	116
14SerH	61	0.8	98	58	2.7	204
14SerHB2	110	2.2	103	88	2.3	435
15GlyH	239	2.1	104	99	1.7	305

16ArgH	183	2.5	105	54	2.6	255
16ArgHD3	325	2.6	103	205	2.5	228
16ArgHE	241	2.4	107	171	3.4	241
17ProHD2	430	2.5	109	145	1.3	144
19ProQG	607	7	92	252	1.6	88
18ProHA	52	1	104	48	2.1	324
19ProHA	149	3	118	262	3.8	152
20SerH	129	2.2	118	246	2.4	158

[a] Average duration of contacts longer than 60 ps.

VI. Correlation of Shell 1 Occupancy and Number of F or Water O Atom Contacts

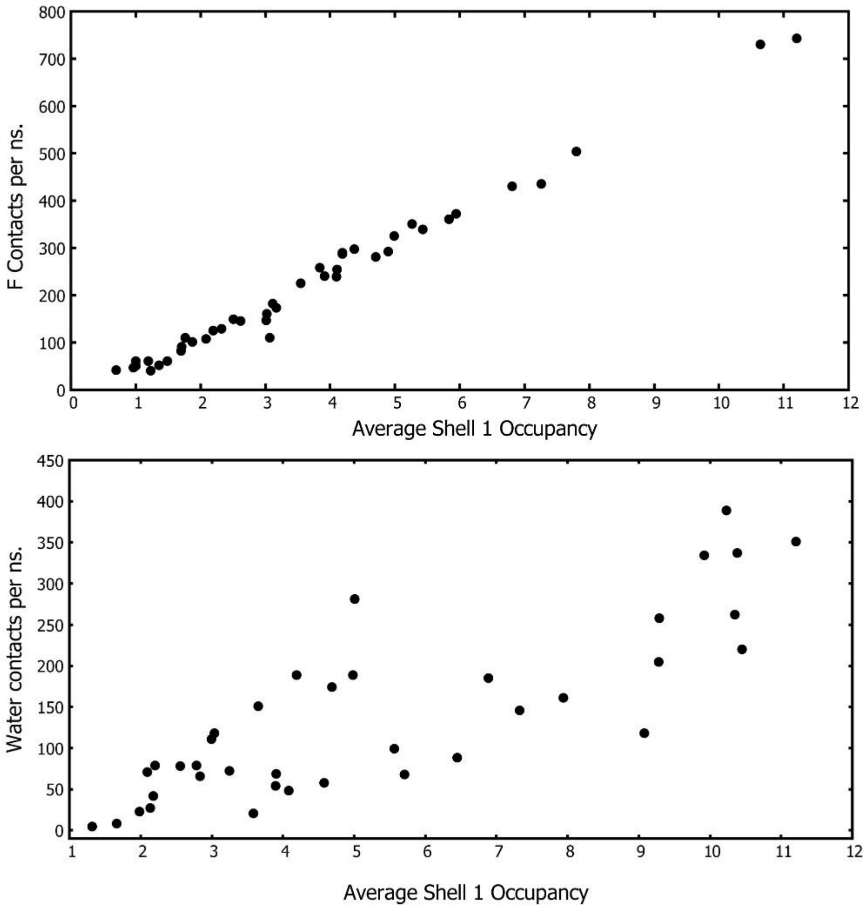

Solvent shell 1 is defined in Figure 1 of the main paper. The average number of contacts with fluorine atoms of solvent HFIP molecules is given. Water contacts were based on oxygen atom contacts. Results for at least 4 simulations of duration 0.2 μs were averaged for each data point.

VII. Calculated Σ_{HF} Parameters for Hydrogen Atoms of Trp-cage Interacting with HFIP in 28%HFIP-water at 298 K

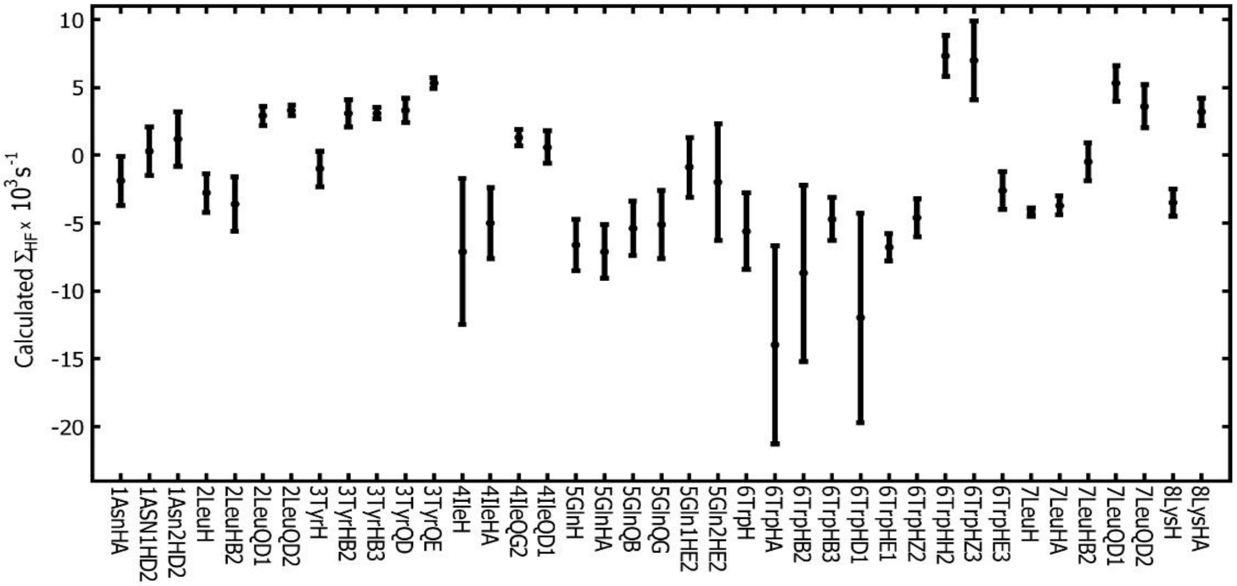

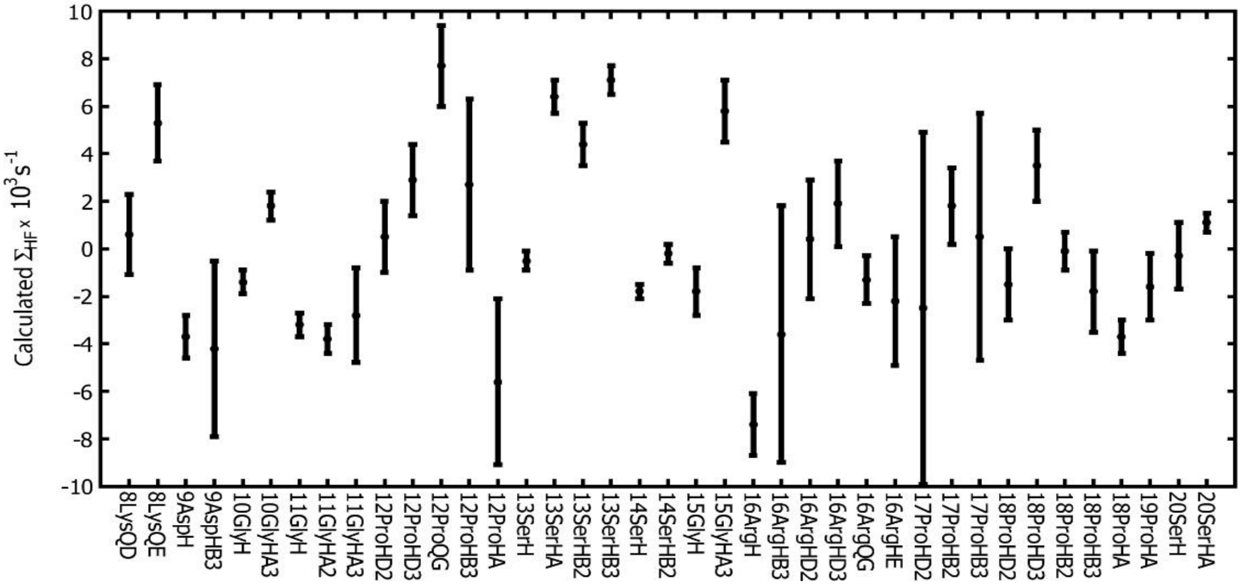

Average results from 7 or more independent simulations of Trp-cage in 28% HFIP-water at 298 K which ranged in duration from 0.2 μs to 0.6 μs are shown. The error bars represent ± the computed mean deviation of Σ_{HF}. Many experimental values of Σ_{HF} are close to zero; see Figure 1 in the main paper.

REFERENCES

1. Hornak, V.; Abel, R.; Okur, A.; Stockbine, B.; Roitberg, A.; Simmerling, C., Comparison of multiple AMBER force fields and development of improved protein backbone parameters. *Proteins: Struct., Func., Bioinf.* **2006,** *65*, 712-725.
2. Lindorff-Larsen, K.; Piana, S.; Palmo, K.; Maragakis, P.; Klepeis, J. L.; Dror, R. O.; Shaw, D. E., Improved side-chain torsion potentials for the Amber ff99SB protein force field. *Prot. Struct. Funct. Bioinfo.* **2010,** *1950-1958*.
3. Neidigh, J. W.; Fesinmeyer, R. M.; Andersen, N. H., Designing a 20-residue protein. *Nat. Struct. Mol. Biol.* **2002,** *9*, 425-430.
4. Chatterjee, C.; Gerig, J. T., Interactions of hexafluoro-2-propanol with Trp-cage peptide. *Biochemistry* **2006,** *45*, 14665-14674.
